# “Inside the Gut–Brain Axis”: Psychological Profiles of Adolescents with Inflammatory Bowel Diseases and with Restrictive Eating Disorders

**DOI:** 10.3390/nu17101706

**Published:** 2025-05-17

**Authors:** Anna Riva, Gabriele Arienti, Giovanna Zuin, Laura Spini, Naire Sansotta, Andrea Eugenio Cavanna, Renata Nacinovich

**Affiliations:** 1 Department of Child Neuropsychiatry, Fondazione IRCCS San Gerardo dei Tintori, 20900 Monza, Italy; anna.riva@irccs-sangerardo.it (A.R.); gabriele.arienti@irccs-sangerardo.it (G.A.); l.spini4@campus.unimib.it (L.S.); 2 School of Medicine and Surgery, University of Milano-Bicocca, 20900 Monza, Italy; a.e.cavanna@bham.ac.uk; 3 Department of Pediatrics, Fondazione IRCCS San Gerardo dei Tintori, 20900 Monza, Italy; giovanna.zuin@irccs-sangerardo.it; 4 Department of Paediatric Hepatology Gastroenterology and Transplantation, Papa Giovanni XXIII Hospital, 24127 Bergamo, Italy; nsansotta@asst-pg23.it; 5 Department of Neuropsychiatry, National Centre for Mental Health, Birmingham and Solihull Mental Health NHS Foundation Trust, Birmingham B15 2FG, UK; 6 School of Medical Sciences, College of Medicine and Health, University of Birmingham, Birmingham B15 2TT, UK; 7 School of Life and Health Sciences, Aston Brain Centre, Aston University, Birmingham B4 7ET, UK; 8 Sobell Department of Motor Neuroscience and Movement Disorders, Institute of Neurology, University College London, London WC1N 3BG, UK

**Keywords:** gut–brain axis, inflammatory bowel diseases, restrictive eating disorders, adolescents, psychological symptoms

## Abstract

**Background**: Individuals with inflammatory bowel diseases (IBDs) have an increased risk of developing psychiatric co-morbidities, including restrictive eating disorders (REDs), with which they share common pathogenic mechanisms, including gut–brain axis dysregulation. We conducted a case–control study systematically exploring the psychopathological profiles and alexithymia in adolescents with IBDs compared with a clinical group of adolescents diagnosed with REDs in order to test the hypothesis of common psychological characteristics between the two patient populations. **Methods**: We recruited 76 patients with IBDs and 76 age-matched controls with REDs (64 adolescents with anorexia nervosa and 12 adolescents with avoidant/restrictive food intake disorder). All participants completed a validated psychometric battery assessing psychological symptoms (SCL-90-R), ED features (EDI-3), and alexithymia (TAS-20). Comprehensive socio-demographic and clinical data were extracted from the medical records. **Results**: A total of 12 patients with IBDs (15.8%) scored higher than the cut-off (>70th percentile) on the EDI-3 scale for Eating Disorder Risk (EDI-EDRC), with a psychological profile comparable to RED patients. Female gender (OR = 0.133, *p* = 0.020) and longer disease duration (OR = 1.055, *p* = 0.036) were identified as significant risk factors for the development of EDs. **Conclusions**: Our findings suggest common psychological traits between patients with REDs and patients with IBDs at risk of developing EDs during adolescence, highlighting the need for early screening for EDs in patients with IBDs who present with specific socio-demographic and disease characteristics.

## 1. Introduction

Inflammatory bowel diseases (IBDs) are chronic, relapsing inflammatory disorders of the gastrointestinal tract, with an etiology that remains incompletely understood. These conditions typically arise in individuals with a genetic predisposition, with onset in childhood in approximately 25% of cases [1]. The spectrum of IBDs includes Crohn’s disease (CD), ulcerative colitis (UC), and IBD unclassified (IBD-U) [2]. In recent years, an increasing number of studies [3,4,5,6] have reported an association between gastrointestinal diseases, including IBDs, and various psychiatric disorders, raising the possibility of extensive genetic correlations and clinical overlaps [7]. This has led to the formulation of the concept of the “gut–brain axis”, which encapsulates the complex bidirectional communication network between the central nervous system and the gastrointestinal system. Specifically, dysregulation of the gut–brain axis appears to be involved in the pathogenesis of IBDs [8], which have long been associated with mental conditions such as stress, anxiety, and depression, as well as in the physiopathology of neurodevelopmental, neurodegenerative, and psychiatric disorders [9].

Although psychological problems are a significant aspect of morbidity and reduced quality of life in patients with IBDs [10], co-morbid psychiatric disorders are often neither recognized nor treated in these individuals [11,12]. For example, the study conducted by Marafini et al. [12] on 237 adult patients with IBDs revealed that 48% had at least one psychiatric diagnosis (predominantly mood and anxiety disorders), with nearly 60% of these diagnoses having been formulated for the first time during the clinical evaluation for the actual study. Psychological vulnerabilities and psychiatric co-morbidities often affect individuals with pediatric-onset IBD. In a recent case–control study on a sample of 52 adolescents with IBDs vs. 52 healthy controls, it was observed that adolescents with IBDs reported significantly higher rates of psychological distress than their peers, reporting more severe symptoms across all areas of psychopathology, with the exception of alexithymia [13]. The results of international cohort studies based on pediatric populations in the United States [5], Sweden [14], and the United Kingdom [6] confirmed that the diagnosis of IBD is associated with an increased risk of developing psychiatric disorders, including affective and anxiety disorders, post-traumatic stress disorder, self-harm, suicide attempts, sleep disorders, and eating disorders (EDs).

It has been suggested that EDs might share common pathogenic mechanisms with IBDs [15]. In particular, it has been pointed out that these patient populations share both genetic and environmental factors, as well as psychological issues and misconceptions about the effects of diet on the exacerbation of gastrointestinal symptoms. This often leads to the implementation of dietary changes and restrictive eating behaviours [16,17]. Indeed, patients with IBDs might associate the worsening of gastrointestinal symptoms with specific eating habits, leading to the avoidance of certain foods and a significant reduction in caloric intake [17]. Interestingly, a prospective study conducted by Limdi et al. [18] on 400 adult patients with IBDs found that almost 50% of them believe that diet can trigger IBD, and 57% think it may cause a relapse: as a result of these misconceptions, two-thirds of patients refrained from eating their preferred foods in the hope of preventing disease flare-ups. Unsurprisingly, a number of symptomatic features, including lack of appetite, weight loss, malnutrition, diarrhea, and constipation, are reported in the context of both IBDs and EDs.

Despite the available evidence and the increased interest in the study of the overlap between IBDs and restrictive eating disorders (REDs) [19], to the best of our knowledge, no study has been conducted to compare the psychological profiles of these two patient populations. Furthermore, predicting factors for the development of EDs in individuals with IBD, particularly in pediatric populations, remain poorly investigated. We therefore set out to conduct a clinical study with a two-fold aim. The primary objective of our study was to compare the psychological profile of adolescents with IBD to that of age-matched adolescents with REDs (anorexia nervosa (AN), atypical anorexia nervosa (AAN), avoidant/restrictive food intake disorder (ARFID), and other non-specified eating disorders with restrictive characteristics (OSFED)) [20], in order to test the hypothesis that patients with REDs and patients with IBDs at risk of developing EDs share common psychological characteristics. Our secondary objective was to identify potential predictors for the development of EDs within the socio-demographic and clinical characteristics—such as female sex, longer disease duration, and exposure to steroid cycles—of patients with IBDs, in order to facilitate the early detection of subjects at risk of developing EDs.

## 2. Materials and Methods

### 2.1. Clinical Samples

Between October 2022 and April 2024, we recruited a total of 168 adolescents aged between 13 and 18 years, from two different cohorts:

- IBDs cohort: Eighty-four patients with a diagnosis of inflammatory bowel disease from at least 6 months, confirmed by a combination of endoscopic, radiological, biochemical, and histological investigations, according to the ESPGHAN Revised Porto Criteria, recruited from the Pediatric Gastroenterology Unit, Department of Pediatrics, Fondazione IRCCS San Gerardo dei Tintori (Monza, Italy) and from the Department of Pediatric Hepatology Gastroenterology and Transplantation, Papa Giovanni XXIII Hospital (Bergamo, Italy);
- REDs cohort: Eighty-four adolescents with a DSM-5-TR-validated diagnosis of REDs [20] recruited from the Department of Child Neuropsychiatry, Fondazione IRCCS San Gerardo dei Tintori (Monza, Italy), assessed by means of the semi-structured DSM-5 interview [21].

The exclusion criteria for patient recruitment were as follows: a diagnosis of psychiatric co-morbidities (including EDs) in patients with IBDs, intellectual disabilities, insufficient proficiency in the Italian language, and substance/alcohol abuse.

**Figure 1:**
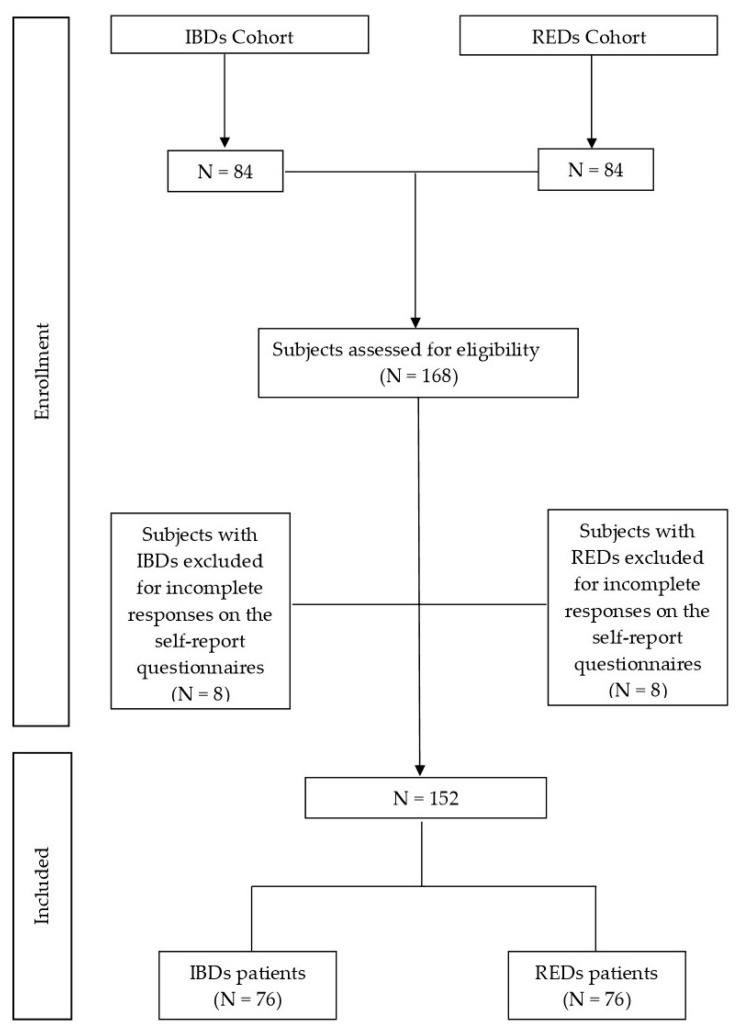
Flow diagram of study cohort selection. Abbreviations: IBDs, inflammatory bowel diseases; REDs, restrictive eating disorders; N, number.

A total of 8 patients with IBDs and 8 patients with REDs were excluded due to incomplete responses on the self-report questionnaire (see Figure 1). Therefore, the final study cohort consisted of 76 patients in each group (152 patients in total).

All adolescents and their parents were provided with detailed information about the purpose of the study, and written informed consent was obtained from the participants’ parents. The study received ethical approval from the Brianza Ethics Committee (Protocol code: 311, 29 June 2023), in accordance with the principles of the Declaration of Helsinki (1964) and its subsequent amendments.

### 2.2. Measures

An ad hoc questionnaire was designed to systematically collect both demographic data (gender, age at diagnosis and at the time of evaluation; family socio-economic status based on the Hollingshead index [22]) and disease-related clinical characteristics (Body Mass Index -BMI- at diagnosis and at the time of evaluation, disease duration expressed in months and type of IBD). Additionally, the following clinical characteristics were collected from the IBD group: disease localization/extent according to the Paris Classification [23]; diagnostic delay, defined as the months elapsed between the onset of symptoms and the diagnosis; disease activity at evaluation, assessed using the Pediatric Crohn Disease Activity Index (PCDAI) for CD [24] and the Pediatric Ulcerative Colitis Activity Index (PUCAI) for UC [25]; therapy at the time of evaluation; number of total relapses (both during the entire course of disease and in the previous six months); number of steroid cycles (both during the entire course of disease and in the previous six months); and number of hospital admissions.

In addition to providing socio-demographic and clinical data, all participants completed a psychometric battery comprising the Italian versions of validated measures of psychological symptoms [26], alexithymia [27], and eating symptomatology [28].

The Symptom Checklist-90–Revised (SCL-90-R) is a self-report questionnaire designed to assess psychological problems and psychopathological symptoms in individuals aged 12 years and older. The SCL-90-R consists of 90 items rated on a 5-point Likert scale that assess nine symptom dimensions: somatization (SOM), obsessive-compulsive (O-C), interpersonal sensitivity (I-S), depression (DEP), anxiety (ANX), hostility (HOS), phobic anxiety (PHOB), paranoid ideation (PAR), psychoticism (PSY). The SCL-90-R includes a Global Severity Index (GSI), which quantifies the risk of developing psychiatric disorders: scores between 55 and 65 are considered borderline, whereas scores higher than 65 are considered pathological. The Italian version of the SCL-90-R showed good internal coherence for all subscales (α values between 0.70 and 0.96) [26].

The Toronto Alexithymia Scale-20 (TAS-20) is a 5-point Likert-type self-report questionnaire consisting of 20 items that assess alexithymia across three factors: Difficulty Identifying Feelings (DIF), Difficulty Describing Feelings (DDF), and Lack of Focus on Internal Emotional Experiences (EOT). A total score of at least 61 indicates the presence of alexithymia. The Italian version of the TAS-20 is characterized by good internal consistency (Cronbach’s α 0.75 and 0.82 in normal and clinical groups, respectively) and high test–retest reliability over 2 weeks (r = 0.86). A confirmatory factor analysis revealed the same factor structure as the original English version and adequate internal consistency of the subscales, with α coefficients equal to or greater than 0.70 [27].

The Eating Disorders Inventory-3 (EDI-3) is a self-report tool measuring psychological traits relevant to individuals with EDs. It consists of 91 items organized into 12 primary scales: three ED-specific scales (Drive for Thinness, Bulimia, Body Dissatisfaction) and nine general psychological scales (Low Self-Esteem, Personal Alienation, Interpersonal Insecurity, Interpersonal Alienation, Interoceptive Deficits, Emotional Dysregulation, Perfectionism, Asceticism, and Maturity Fears) that are highly relevant to, but not specific for, EDs. The primary scales yield six composite scores: one ED-specific risk score (Eating Disorder Risk), and five scales that tap into general integrative psychological constructs (Ineffectiveness, Interpersonal Problems, Affective Problems, Overcontrol, and Global Psychological Maladjustment). The Italian version of the EDI-3 demonstrated high internal reliability coefficients (0.80 to 0.90) and test–retest reliability coefficients (0.93 to 0.98) [28].

### 2.3. Data Analysis

Since data were not normally distributed, continuous variables were presented as medians and skewness coefficients (with means and standard deviations reported in the Supplementary Tables S1–S6), whereas categorical variables were presented as absolute or percent frequencies. The Chi-square test or Fisher’s exact test (for frequencies below 5) was used to compare categorical variables between groups.

Within the IBD cohort, individuals who scored above the 70th percentile on the EDI-3 EDRC subscale (cut-off value for pathological scores) were selected for comparisons with individuals who scored below the cut-off and individuals with REDs. All comparisons of socio-demographic and disease characteristics among the three clinical groups (IBD-EDRC > 70 vs. IBD-EDRC < 70 vs. REDs) were carried out through univariate analysis using the non-parametric Kruskal–Wallis test for multiple comparisons. Since the three groups differed for age at evaluation, BMI at evaluation, gender, and socioeconomic status (SES), a non-parametric ANCOVA with these variables as covariates was used to compare the three groups for EDI-3, TAS-20, and SCL-90-R scores. Finally, a multivariate model of logistic regression analyses was conducted with IBD-EDRC > 70 as the dependent variable and sociodemographic and clinical disease characteristics as independent variables. This was preceded by a Lasso regression to select the most valuable variables to insert in the multivariate model and to exclude possible collinearity between variables.

A significance level of *p* ≤ 0.05 was set for all analyses. These statistical analyses were performed using the Statistical Package for the Social Sciences (SPSS, version 29.0.1.0; IBM Corp., Armonk, NY, USA).

## 3. Results

We analyzed data from 152 adolescents aged 13 to 18 years: 76 adolescents affected by IBDs and 76 adolescents affected by REDs. The socio-demographic and clinical characteristics of the two groups are summarized in Table 1. In the group of adolescents with IBD, the diagnosis of UC was the most frequent (61.8%), followed by CD (35.5%), and unclassified IBD (IBD-U) (2.6%). In the group of adolescents affected by REDs, 84.2% (64/76) received a diagnosis of AN, whereas 15.8% (12/76) were diagnosed with ARFID, according to DSM-5-TR criteria [20]. Patients with IBDs and patients with REDs differed for gender (*p* < 0.001), socio-economic status (*p* < 0.001), BMI at diagnosis (*p* < 0.001), and BMI at evaluation (*p* < 0.001).

The disease characteristics of subjects affected by IBD are reported in Table 2. At the time of evaluation, 57/74 (77.0%, diagnosed with either UC or CD) were in clinical remission.

Within the IBD cohort, we examined how many of them were at risk of developing EDs based on the score obtained on the composite EDRC subscale of the EDI-3 test. As many as 12/76 patients with IBDs (15.8%, seven subjects with UC and five with CD) were found to be at risk of developing EDs, as they scored above the recommended cut-off (EDI-EDRC > 70th percentile).

Table 3 shows the results of the comparison analyses of socio-demographic and clinical disease characteristics among the three clinical groups: Group 1 (IBD-EDRC > 70), Group 2 (IBD-EDRC < 70), and Group 3 (REDs). The three groups differed significantly from each other in gender ratio, as females represented 75% of subjects in Group 1, 37.5% in Group 2, and 96% in Group 3 (test 1–2: *p* = 0.018; test 2–3: *p* < 0.001; test 1–3: *p* = 0.031). Groups 1 and 2 were comparable for all continuous variables considered, with no statistically significant differences. Group 1 and Group 3 were comparable in terms of socio-economic status (*p* = 0.088), age at evaluation (*p* = 0.524), and age at diagnosis (*p* = 0.175). However, they differed significantly in terms of BMI at the time of evaluation (*p* < 0.001), BMI at diagnosis (*p* < 0.001), and disease duration (*p* < 0.001). Finally, Group 2 was found to be comparable to Group 3 only in terms of age at evaluation (*p* = 0.070).

Since the three groups differed for age at evaluation, BMI at evaluation, gender, and SES, a non-parametric ANCOVA with these variables as covariates was used to compare the results of the psychometric tests of the three groups. In particular, since SES can influence both mental health outcomes and disease management, the adjusted ANCOVA fully accounted for this potential confounder.

The results of the comparison between the three groups with regard to the EDI-3 test are summarized in Table 4. Patients in Group 1 and patients in Group 3 showed statistically significant differences in selected EDI-3 subscales, namely Bulimia (*p* = 0.023), Emotional Dysregulation (*p* = 0.035), Perfectionism (*p* = 0.023), Affective Problems Composite (*p* = 0.035), and Overcontrol Composite (*p* = 0.009). In contrast, patients in Group 2 reported statistically significant differences across all subscales when compared to both Group 1 (except for Interpersonal Insecurity, *p* = 0.102, and for Interpersonal Problems, *p* = 0.110) and Group 3 (except for Bulimia, *p* = 0.082). Patients in Group 3 scored above the clinically relevant cut-offs across all EDI-3 subscales, except for the Bulimia subscale (B), where the median score was 61. Group 1, on the other hand, reported median scores above the recommended cut-offs on the following subscales: Drive for Thinness (median 73), Interpersonal Insecurity (median 73), Interoceptive Deficits (median 78), Eating Disorder Risk Composite (median 72), Interpersonal Problems (median 70), Affective Problems Composite (median 74), and Global Psychological Maladjustment Composite (median 73). Finally, Group 1 displayed median scores in the individual subscales that were higher than Group 2 but lower than Group 3, all of which were below the clinically relevant thresholds.

The results of the comparisons for the TAS-20 scale are reported in Table 5. Group 1 and Group 3 did not show statistically significant differences in TAS-20 TOTAL (*p* = 0.841), DIF (*p* = 0.290), DDF (*p* = 0.797), or EOT scores (*p* = 0.528). Group 2, however, reported statistically significant differences with the other two groups in the TAS-20 TOTAL score (*p* = 0.031 with Group 1 and *p* < 0.001 with Group 3), as well as in DIF (*p* < 0.001) and DDF scores (*p* = 0.004) when compared to Group 3. Patients in Group 3 reported a median TAS-20 score of 66, which is above the threshold for the risk of developing alexithymia. In contrast, patients in Group 1 and Group 2 scored below the threshold value (58 and 54, respectively).

The results of the comparisons for the SCL-90-R questionnaire are summarized in Table 6. Group 1 and Group 3 did not show statistically significant differences in any of the subscales, except for the somatization scale (*p* = 0.047), where patients in Group 1 reported lower scores. Patients in Group 1 and Group 2 reported statistically significant differences on all subscales, except for the phobic anxiety dimension (*p* = 0.050). Likewise, Group 2 differed significantly from Group 3 in all subscales except for the hostility dimension (*p* = 0.074). Patients in Group 1 reported intermediate scores between Group 2 and Group 3 across all SCL-90-R subscales.

The main socio-demographic (gender, socio-economic status, age at diagnosis) and clinical characteristics (BMI at diagnosis and BMI at evaluation, disease duration, diagnostic delay, number of relapses, number of relapses in the last 6 months, number of steroid cycles, number of steroid cycles in the last 6 months, number of hospital admissions) were included in a Lasso regression to select the most relevant variables. Both BMI at evaluation and number of steroid cycles in the last 6 months were excluded from the multivariate model of logistic regression analyses as non-relevant.

As reported in Table 7, the selected socio-demographic and clinical disease characteristics were included in a logistic regression model using IBD-EDRC > 70 as the dependent variable. The model resulted was statistically valid (B = −1.642, *p* < 0.001). Two variables were identified as significant predictors for the risk of developing EDs in individuals with IBDs: female gender (*p* = 0.020) and longer disease duration (*p* = 0.036).

## 4. Discussion

Individuals with IBDs, including children and adolescents, have an increased risk of developing psychiatric disorders [5,6,14], including EDs, which are thought to share common pathogenic mechanisms with gastrointestinal diseases [15]. Although the intersection between EDs and gastrointestinal diseases has been a topic of increasing interest in the medical literature [29], to the best of our knowledge, this study represents the first effort to compare the psychological profile of adolescents with IBDs to that of adolescents with REDs, based on the hypothesis of shared psychological characteristics.

More than half of our cohort (61.8%) with IBDs consisted of subjects with UC. These findings align with epidemiological data reported in the scientific literature: unlike in other European countries, in Italy, the number of UC diagnoses in the pediatric population exceeds that of CD (49.5% vs. 43.1%) [30]. At the time of evaluation, the majority (77%) of subjects with IBDs presented with a condition in clinical remission (assessed using the PCDAI/PUCAI scores [24,25]).

Overall, 16% of our IBD sample screened positive for the risk of developing EDs, reporting scores above the pathological cut-off (>70th percentile) on the EDRC subscale of the EDI-3 test. This result, which is approximately six times higher than the 2.7% [31] risk of developing an ED in the general adolescent population, aligns with the existing literature, which suggests that the risk of developing an ED in patients with IBDs ranges from 5% to 17%, depending on the age of the sample and the type of ED, suggesting a strong correlation between IBDs and EDs [16,32]. Similarly, the above-mentioned result is consistent with the risk of EDs in other gastrointestinal conditions. A recent narrative review [29] reported an elevated prevalence of EDs—ranging from 15.7% to 18%—including ARFID, among individuals with coeliac disease across multiple cohorts. Similarly, adolescents with irritable bowel syndrome (IBS) have been found to engage more frequently than healthy controls in disordered eating behaviours such as avoiding trigger foods, not eating when hungry, and vomiting after eating. However, findings remain inconsistent: a large study of 228 adults found no significant increase in disordered eating behaviours among individuals with IBS compared to controls. Notably, several factors have been associated with increased ED risk in IBS, including symptom severity and duration, female sex, younger age, and the presence of anxiety and depression. The comparison of EDI-3, TAS-20, and SCL-90-R scores between the EDI-EDRC < 70 group, the EDI-EDRC > 70 group, and the REDs group showed that individuals with IBD-EDRC > 70, and thus potentially at risk of developing an ED, were characterized by a psychopathological profile almost completely overlapping with that of patients with confirmed REDs, and significantly different from the IBD-EDRC < 70 group. In particular, the EDI-EDRC > 70 and REDs group reported similar scores in key areas (Drive For Thinness, Interpersonal Insecurity and Alienation, Interoceptive Deficits, Affective Problems, anxiety, depression, interpersonal sensitivity, and alexithymia) as assessed by the EDI-3, TAS-20 and SCL-90-R measures, which have been identified as core features for the development of EDs [33,34,35]. These results are consistent with findings reported in several clinical studies, according to which patients with IBDs present with multiple characteristics identified as risk factors for EDs, including concurrent anxiety and depression, body image dissatisfaction, and avoidance of social settings [36].

The secondary findings of our study allowed us to identify potential risk factors for the development of EDs within the socio-demographic and clinical characteristics of adolescents with IBD—a clinically relevant but relatively under—investigated area [37]. Our results indicated that female sex and longer disease duration are associated with an increased risk for the development of EDs. Female gender is a well-established general risk factor for developing EDs, not only in patients with IBD [36,38], due to both psychosocial and hormonal factors [39]. Adolescence, a period of significant biological and psychosocial changes, has been identified as a particularly risky time for the development of EDs, which peak in incidence and prevalence during this life stage [33,36]. Consistent with previous studies [32,40], longer disease duration appeared to be a significant predictor of the risk of developing EDs, as it may be associated with the emergence of alexithymic traits, which are themselves implicated in the development and maintenance of EDs [41,42].

Although arguably innovative, our study has several limitations. First, the relatively small sample size resulted in reduced statistical power, possibly limiting the ability to detect significant associations with certain disease characteristics and to generalize our results. A second limitation consisted of the use of self-assessment tests in a population characterized by alterations in self-awareness (both healthy adolescents and clinical populations). Furthermore, regarding the study’s limitations, the absence of a control group of healthy adolescents prevents a direct comparison between patients with IBDs and their healthy peers. Finally, there may be a referral bias related to the recruitment of patients with either EDs or IBDs from tertiary care centres, where patients with more complex and/or severe clinical pictures are seen. Specifically, when compared to epidemiological data from the years 2009–2018 collected through the pediatric inflammatory bowel diseases (PIBD) registry [30], our IBD cohort displayed greater disease severity, as evidenced by shorter diagnostic delay and earlier and more frequent use of biologic therapies. Of note, our clinical sample and the PIBD registry showed a similar disease distribution—predominantly ileocolonic in patients with CD and pancolitis in patients with UC. As a result of this, our study population may not be fully representative of the entire population of adolescents with REDs or IBDs.

## 5. Conclusions

We found that the psychopathological profile of adolescents with IBDs overlaps to a significant degree with that of patients with a confirmed diagnosis of REDs. If confirmed, these results highlight the need for greater attention and routine screening for the development of EDs in adolescents with IBDs, particularly in association with certain clinical and socio-demographic characteristics. Therefore, it seems appropriate for these patients to receive multidisciplinary input, with the early involvement of pediatric gastroenterologists, child and adolescent psychiatrists, dietitians, and psychologists.

Future research should focus on identifying new and effective prevention and intervention strategies for patients with IBD at risk of developing EDs, in order to avoid misdiagnosis/delayed diagnosis and/or inappropriate treatments, especially during the developmental age.

## Figures and Tables

**Table 1:** Socio-demographical and clinical characteristics of patients with IBDs and patients with REDs.

	IBDs (N = 76)	REDs (N = 76)	*p*-Value
Female, N (%)	33 (43.4)	73 (96.0)	<0.001 **
Age at evaluation, median (skewness)	16.00 (−0.423)	16.00 (−0.341)	0.161
Socio-economic status, median (skewness)	29.00 (0.577)	37.00 (0.049)	<0.001 **
BMI at evaluation, median (skewness)	20.67 (0.489)	17.00 (0.585)	<0.001 **
BMI at diagnosis, median (skewness)	17.74 (0.647)	15.80 (0.786)	<0.001 **
Disease duration (months), median (skewness)	33.00 (1.562)	13.00 (1.019)	<0.001 **

Abbreviations: IBD, inflammatory bowel disease; REDs, restrictive eating disorders; BMI, Body Mass Index; N, number. ** *p* ≤ 0.001.

**Table 2:** Clinical disease characteristics of patients with IBDs (N = 76).

Diagnosis, N (%)	
CD	27 (35.5)
UC	47 (61.8)
IBD-U	2 (2.6)
Clinical Disease Activity at evaluation, N (%)	
Remission	57 (77.0)
Mild	15 (20.3)
Moderate-severe	2 (2.7)
Diagnostic delay (months),	
median (skewness)	3.0 (1.773)
Pharmacological therapy, N (%)	
Biological drugs	46 (62.2)
Polytherapy	30 (40.5)
Number of steroid cycles,	
median (skewness)	1.0 (1.433)
Number of relapses,	
median (skewness)	1.0 (1.343)
Number of relapses in the previous 6 months,	
median (skewness)	0.0 (3.644)
Number of hospital admissions,	
median (skewness)	1.0 (1.307)

Abbreviations: UC, ulcerative colitis; CD, Crohn’s disease; IBD-U, inflammatory bowel disease unclassified.

**Table 3:** Comparison of socio-demographic and clinical characteristics among the three clinical groups (IBD-EDRC > 70 vs. IBD-EDRC < 70 vs. REDs).

	GROUP 1 IBD-EDRC > 70 (N = 12)	GROUP 2 IBD-EDRC < 70 (N = 64)	GROUP 3 REDs (N = 76)	Test 1–2 *p*-Value	Test 2–3 *p*-Value	Test 1–3 *p*-Value
Female gender, N (%)	9 (75.0)	24 (37.5)	73 (96.0)	0.018 *	<0.001 **	0.031 *
Socio-economic status, median (skewness)	32 (0.730)	29 (0.571)	37 (0.049)	0.931	<0.001 **	0.088
Age at evaluation, median (skewness)	16 (0.539)	16 (−0.323)	16 (−0.341)	0.109	0.070	0.524
Age at diagnosis, median (skewness)	13 (−1.306)	13 (−1.195)	15 (−0.373)	0.573	<0.001 **	0.175
BMI at evaluation, median (skewness)	23 (−0.230)	20 (0.616)	17 (0.585)	0.153	<0.001 **	<0.001 **
BMI at diagnosis, median (skewness)	19 (0.776)	17 (0.567)	16 (0.786)	0.076	<0.001 **	<0.001 **
Disease duration (months), median (skewness)	48 (0.861)	33 (1.783)	13 (1.019)	0.404	<0.001 **	<0.001 **

Abbreviations: IBD, inflammatory bowel disease; EDRC, Eating Disorder Risk Composite; REDs, restrictive eating disorders; BMI, Body Mass Index. * p ≤ 0.05, ** *p* ≤ 0.001.

**Table 4:** Comparison of EDI-3 results between the three clinical groups (IBD-EDRC > 70 vs. IBD-EDRC < 70 vs. REDs) adjusted for age at evaluation, BMI at evaluation, gender, and socio-economic status.

	GROUP 1 IBD-EDRC > 70		GROUP 2 IBD-EDRC < 70		GROUP 3 REDs		Test 1–2	Test 2–3	Test 1–3
	Median	Skewness	Median	Skewness	Median	Skewness	*p*-Value	*p*-Value	*p*-Value
EDI-DT	73	−0.436	34	0.076	87	−1.855	0.001 *	<0.001 **	0.404
EDI-B	61	−0.421	42	0.137	61	−0.309	0.002 *	0.082	0.023 *
EDI-BD	64	−0.219	29	0.533	85	−1.249	<0.001 **	<0.001 **	0.072
EDI-LSE	67	−0.527	47	−0.027	85	−1.431	0.001 *	<0.001 **	0.486
EDI-PA	65	−0.495	44	−0.006	86	−1.246	<0.001 **	<0.001 **	0.160
EDI-II	73	−0.640	60	−0.318	87	−1.462	0.102	<0.001 **	0.850
EDI-IA	59	−0.356	45	0.068	74	−0.742	0.196	0.029 *	0.891
EDI-ID	78	−0.646	51	−0.075	88	−1.097	<0.001 **	<0.001 **	0.085
EDI-ED	64	−0.509	50	−0.049	74	−0.794	0.002 *	0.066	0.035 *
EDI-P	59	−0.236	41	0.183	74	−0.518	<0.001 **	0.028 *	0.023 *
EDI-A	69	−0.680	50	−0.193	80	−1.064	<0.001 **	0.001 *	0.053
EDI-MF	59	−0.252	53	−0.074	73	−0.517	0.463	0.021 *	0.618
EDI-IC	69	−0.516	50	0.010	86	−1.585	<0.001 **	<0.001 **	0.316
EDI-IPC	70	−0.583	57	−0.276	85	−1.234	0.110	0.020 *	0.927
EDI-APC	74	−0.625	52	−0.028	82	−1.068	0.001 *	<0.001 **	0.035 *
EDI-OC	65	−0.511	46	0.070	80	−1.118	<0.001 **	0.001 *	0.009 *
EDI-GPMC	73	−0.647	58	−0.215	87	−1.681	<0.001 **	<0.001 **	0.072

Abbreviations: IBD, inflammatory bowel disease; EDRC, Eating Disorder Risk Composite; REDs, restrictive eating disorders; DT, Drive for Thinness; B, Bulimia; BD, Body Dissatisfaction; LSE, Low Self Esteem; PA, Personal Alienation; II, Interpersonal Insecurity; IA, Interpersonal alienation; ID, Interoceptive Deficits; ED, Emotional Dysregulation; P, Perfectionism; A, Ascetism; MF, Maturity Fear; IC, Ineffectiveness Composite; IPC, Interpersonal Problems; APC, Affective Problems Composite; OC, Overcontrol Composite; GPMC, Global Psychological Maladjustment Composite. * *p* < 0.05, ** *p* ≤ 0.001.

**Table 5:** Comparison of TAS-20 results among the three clinical groups (IBD-EDRC > 70 vs. IBD-EDRC < 70 vs. REDs) adjusted for age at evaluation, BMI at evaluation, gender, and socio-economic status.

	GROUP 1 IBD-EDRC > 70		GROUP 2 IBD-EDRC < 70		GROUP 3 REDs		Test 1–2	Test 2–3	Test 1–3
	Median	Skewness	Median	Skewness	Median	Skewness	*p*-Value	*p*-Value	*p*-Value
TAS-20 TOT	58	−0.170	54	0.298	66	−0.655	0.031 *	<0.001 **	0.841
TAS-20 DIF	22	−0.097	17	0.447	25	−0.584	0.002	<0.001 **	0.290
TAS-20 DDF	17	−0.312	15	−0.054	20	−0.688	0.074	0.004 *	0.797
TAS-20 EOT	20	0.086	20	0.054	21	−0.039	0.866	0.389	0.528

Abbreviations: IBD, inflammatory bowel disease; EDRC, Eating Disorder Risk Composite; REDs, restrictive eating disorders; TOT, total; DIF, Difficulty Identifying Feelings; DDF, Difficulty Describing Feelings; EOT, Lack of Focus on Internal Emotional Experiences. * *p* < 0.05, ** *p* ≤ 0.001.

**Table 6:** Comparison of SCL-90-R results among the three clinical groups (IBD-EDRC > 70 vs. IBD-EDRC < 70 vs. REDs) adjusted for age at evaluation, BMI at evaluation, gender, and socio-economic status.

	GROUP 1 IBD-EDRC > 70		GROUP 2 IBD-EDRC < 70		GROUP 3 REDs		Test 1–2	Test 2–3	Test 1–3
	Median	Skewness	Median	Skewness	Median	Skewness	*p*-Value	*p*-Value	*p*-Value
SCL-90-R-SOM	52	0.340	47	0.843	54	0.105	0.001 *	0.005 *	0.047 *
SCL-90-R-O-C	54	0.126	50	0.581	60	−0.433	0.009 *	<0.001 **	0.465
SCL-90-R-I-S	55	0.063	48	0.612	63	−0.484	0.007 *	<0.001 **	0.516
SCL-90-R-DEP	56	−0.042	52	0.415	66	−0.462	0.003 *	<0.001 **	0.328
SCL-90-R-ANX	55	0.191	50	0.751	63	−0.437	0.002 **	<0.001 **	0.210
SCL-90-R-HOS	47	0.689	45	1.098	48	0.632	0.040 *	0.074	0.050
SCL-90-R-PHOB	54	0.511	48	0.947	57	0.074	0.050	0.030 *	0.406
SCL-90-R-PAR	52	0.180	49	0.549	54	0.069	0.017 *	0.027 *	0.214
SCL-90-R-PSY	53	0.046	47	0.954	58	0.055	<0.003 **	<0.001 **	0.381
SCL-90-R-GSI	56	0.092	50	0.676	62	−0.410	0.001 **	<0.001 **	0.155

Abbreviations: IBD, inflammatory bowel disease; EDRC, Eating Disorder Risk Composite; BMI, Body Mass Index; REDs, restrictive eating disorder; SCL-90-R, Symptom Checklist-90–Revised; SOM, somatization; O-C, Obsessive–Compulsive; I-S, interpersonal sensitivity; DEP, depression; ANX, anxiety; HOS, hostility; PHOB, phobic anxiety; PAR, paranoid ideation; PSY, psychoticism; GSI, Global Severity Index. * *p* < 0.05, ** *p* ≤ 0.001.

**Table 7:** Multivariate model of socio-demographic and clinical predictors of the risk of developing eating disorders in patients with IBD-EDRC > 70.

				95% C.I. for EXP(B)	
Predictive Variables	B	*p* Value	OR	Inf	Sup
Gender (F = 0; M = 1)	−2.018	0.020 *	0.133	0.024	0.730
Age at diagnosis	0.433	0.115	1.542	0.900	2.643
BMI at diagnosis	0.232	0.086	1.261	0.968	1.642
Duration disease	0.053	0.036 *	1.055	1.003	1.108
Diagnostic delay	−0.078	0.204	0.925	0.820	1.043
N of relapses	0.154	0.675	1.166	0.569	2.391
N of relapses in the previous 6 months	−1.672	0.293	0.188	0.008	4.248
N of steroid cycles	0.367	0.312	1.443	0.709	2.936
N of hospital admissions	−0.663	0.245	0.516	0.169	1.576
R^2^ 0.247					

Abbreviations: OR, odds ratio; IBD, inflammatory bowel disease; EDRC, Eating Disorder Risk Composite; F, female; M, male; BMI, Body Mass Index; N, number. * *p* < 0.05.

## Data Availability

The data presented in this study are available on request from the corresponding author due to privacy.

## Supplementary Materials

The following supporting information can be downloaded at https://www.mdpi.com/article/10.3390/nu17101706/s1, Table S1: Socio-demographical and clinical characteristics of patients with IBDs and patients with REDs; Table S2: Clinical disease characteristics of patients with IBDs (N = 76); Table S3: Socio-demographic and clinical characteristics among the three clinical groups (IBD-EDRC > 70 vs. IBD-EDRC < 70 vs. REDs); Table S4: Descriptive statistics of EDI-3 results for the three clinical groups (IBD-EDRC > 70 vs. IBD-EDRC < 70 vs. REDs); Table S5: Descriptive statistics of TAS-20 results for the three clinical groups (IBD-EDRC > 70 vs. IBD-EDRC < 70 vs. REDs); Table S6: Descriptive statistics of SCL-90-R results for the three clinical groups (IBD-EDRC > 70 vs. IBD-EDRC < 70 vs. REDs).
