# Supplementary material for: “Inside the Gut–Brain Axis”: Psychological Profiles of Adolescents with Inflammatory Bowel Diseases and with Restrictive Eating Disorders
Source: Nutrients. 2025 May 17;17(10):1706. doi: 10.3390/nu17101706 (PMC12114306; doi:10.3390/nu17101706)
Supplement: Supplementary file 1 [file nutrients-17-01706-s001.zip › nutrients-3610813-supplementary.pdf]

SUPPLEMENTAL TABLES

Table S1. Socio-demographical and clinical characteristics of patients with IBDs and patients with REDs.

	IBDs (N=76)	REDs (N=76)
Female, N (%)	33 (43.4)	73 (96.0)
Age at evaluation, median (skewness)	16.00 (-0.423)	16.00 (-0.341)
mean (SD)	15.51 (1.88)	16.00 (1.60)
Socio-economic status, median (skewness)	29.00 (0.577)	37.00 (0.049)
mean (SD)	31.41 (12.56)	38.54 (11.82)
BMI at evaluation, median (skewness)	20.67 (0.489)	17.00 (0.585)
mean (SD)	20.70 (3.35)	17.23 (2.73)
BMI at diagnosis, median (skewness)	17.74 (0.647)	15.80 (0.786)
mean (SD)	18.19 (3.48)	16.09 (2.60)
Disease duration (months), median (skewness)	33.00 (1.562)	13.00 (1.019)
mean (SD)	43.91(35.76)	15.55 (9.93)

Abbreviations: IBD, Inflammatory Bowel Disease; REDs, restrictive eating disorders; SES, socio-economic status; BMI, Body Mass Index; N, number; SD, standard deviation.

Table S2. Clinical disease characteristics of patients with IBDs (N=76).

Diagnosis, N (%)	
CD	27 (35.5)
UC	47 (61.8)
IBD-U	2 (2.6)
Clinical Disease Activity at evaluation, N (%)	
remission	57 (77.0)
mild	15 (20.3)
moderate-severe	2 (2.7)
Diagnostic delay (months),	
mean (SD)	6.37 (7.20)
median (skewness)	3.0 (1.773)
Pharmacological therapy, N (%)	
biological drugs	46 (62.2)
polytherapy	30 (40.5)
Number of steroid cycles,	
mean (SD)	1.12 (1.34)
median (skewness)	1.0 (1.433)
Number of relapses,	
mean (SD)	1.29 (1.67)
median (skewness)	1.0 (1.343)
Number of relapses in the previous 6 months,	
mean (SD)	0.24 (0.67)
median (skewness)	0.0 (3.644)
Number of hospital admissions,	
mean (SD)	0.95 (1.10)
median (skewness)	1.0 (1.307)

Abbreviations: UC, ulcerative colitis; CD, Crohn's disease; IBD, Inflammatory Bowel Disease; IBD-U, Inflammatory Bowel Disease unclassified; SD, standard deviation.

Table S3. Socio-demographic and clinical characteristics among the three clinical groups (IBD-EDRC>70 vs IBD-EDRC<70 vs REDs).

	GROUP 1 IBD-EDRC>70 (N=12)	GROUP 2 IBD-EDRC<70 (N=64)	GROUP 3 REDs (N=76)
Female gender, N (%)	9 (75.0)	24 (37.5)	73 (96.0)
Socio-economic status,			
mean (SD)	31.64 (12.52)	31.26 (12.67)	38.37 (11.85)
median (skewness)	32 (0.730)	29 (0.571)	37 (0.049)
Age at evaluation,			
mean (SD)	16.18 (1.54)	15.34 (1.88)	15.97 (1.64)
median (skewness)	16 (0.539)	16 (-0.323)	16 (-0.341)
Age at diagnosis,			
mean (SD)	12.18 (4.07)	13.00 (2.60)	14.32 (1.57)
median (skewness)	13 (-1.306)	13 (-1.195)	15 (-0.373)
BMI at evaluation,			
mean (SD)	22.61 (3.82)	20.51 (3.06)	17.30 (2.76)
median (skewness)	23 (-0.230)	20 (0.616)	17 (0.585)
BMI at diagnosis,			
mean (SD)	20.29 (4.00)	18.01 (3.33)	16.15 (2.59)
median (skewness)	19 (0.776)	17 (0.567)	16 (0.786)
Disease duration (months),			
mean (SD)	59.18 (42.11)	38.34 (28.2)	15.47 (9.43)
median (skewness)	48 (0.861)	33 (1.783)	13 (1.019)

Abbreviations: IBD, Inflammatory Bowel Disease; EDRC, Eating Disorder Risk Composite; REDs, Restrictive eating disorders; BMI, Body Mass Index, SD, standard deviation.

Table S4. Descriptive statistics of EDI-3 results for the three clinical groups (IBD-EDRC>70 vs IBD-EDRC<70 vs REDs).

	GROUP 1 IBD-EDRC>70				GROUP 2 IBD-EDRC<70				GROUP 3 REDs			
	mean	SD	median	skew	mean	SD	median	skew	mean	SD	median	skew
EDI-DT	78.4	6.64	73	-0.436	27.1	24.31	34	0.076	78.5	25.29	87	-1.855
EDI-B	77.9	18.43	61	-0.421	37.8	30.63	42	0.137	48.6	33.79	61	-0.309
EDI-BD	81.9	8.87	64	-0.219	25.5	18.18	29	0.533	75.7	21.28	85	-1.249
EDI-LSE	75.5	14.32	67	-0.527	37.3	25.15	47	-0.027	77.9	22.80	85	-1.431
EDI-PA	79.2	14.16	65	-0.495	39.0	25.67	44	-0.006	75.4	24.88	86	-1.246
EDI-II	72.9	20.05	73	-0.640	51.4	23.32	60	-0.318	77.2	25.21	87	-1.462
EDI-IA	62.0	26.87	59	-0.356	42.4	26.23	45	0.068	65.4	26.43	74	-0.742
EDI-ID	84.7	13.79	78	-0.646	44.9	26.56	51	-0.075	79.2	21.22	88	-1.097
EDI-ED	78.1	27.27	64	-0.509	42.8	29.53	50	-0.049	67.0	27.55	74	-0.794
EDI-P	75.7	27.24	59	-0.236	39.4	29.59	41	0.183	63.4	29.23	74	-0.518
EDI-A	78.6	11.97	69	-0.680	43.4	26.96	50	-0.193	73.2	20.79	80	-1.064
EDI-MF	60.0	24.33	59	-0.252	48.2	26.48	53	-0.074	64.9	28.16	73	-0.517
EDI-IC	79.0	12.22	69	-0.516	39.0	24.27	50	0.010	78.2	22.39	86	-1.585
EDI-IPC	71.2	20.46	70	-0.583	48.8	24.14	57	-0.276	75.1	24.86	85	-1.234
EDI-APC	84.6	15.54	74	-0.625	44.7	27.15	52	-0.028	77.2	22.01	82	-1.068
EDI-OC	81.6	13.87	65	-0.511	41.3	26.41	46	0.070	71.9	23.41	80	-1.118
EDI-GPMC	83.1	11.66	73	-0.647	46.4	20.67	58	-0.215	79.7	20.40	87	-1.681

Abbreviations: IBD, Inflammatory Bowel Disease; EDRC, Eating Disorder Risk Composite; REDs, restrictive eating disorders; SD, standard deviation; skew, skewness; DT, Drive for Thinness; B, Bulimia; BD, Body Dissatisfaction; LSE, Low Self Esteem; PA, Personal Alienation; II, Interpersonal Insecurity; IA, Interpersonal alienation; ID, Interoceptive Deficits; ED, Emotional Dysregulation; P, Perfectionism; A, Ascetism; MF, Maturity Fear; IC, Ineffectiveness Composite; IPC, Interpersonal Problems; APC, Affective Problems Composite; OC, Overcontrol Composite; GPMC, Global Psychological Maladjustment.

Table S5. Descriptive statistics of TAS-20 results for the three clinical groups (IBD-EDRC>70 vs IBD-EDRC<70 vs REDs).

	GROUP 1 IBD-EDRC>70				GROUP 2 IBD-EDRC<70				GROUP 3 REDs			
	mean	SD	median	skew	mean	SD	median	skew	mean	SD	median	skew
TAS-20 TOT	61.7	12.39	58	-0.170	49.7	11.73	54	0.298	64.0	11.08	66	-0.655
TAS-20 DIF	25.1	5.19	22	-0.097	15.5	6.22	17	0.447	24.0	6.46	25	-0.584
TAS-20 DDF	18.2	4.18	17	-0.312	13.7	4.64	15	-0.054	19.2	4.35	20	-0.688
TAS-20 EOT	18.4	5.28	20	0.086	20.4	5.29	20	0.054	20.8	4.18	21	-0.039

Abbreviations: IBD, Inflammatory Bowel Disease; SD, standard deviation; skew, skewness; EDRC, Eating Disorder Risk Composite; REDs, restrictive eating disorders; TOT, Total; DIF, Difficulty Identifying Feelings; DDF, Difficulty Describing Feelings; EOT, Lack of Focus on Internal Emotional Experiences.

Table S6. Descriptive statistics of SCL-90-R results for the three clinical groups (IBD-EDRC>70 vs IBD-EDRC<70 vs REDs).

	GROUP 1 IBD-EDRC>70				GROUP 2 IBD-EDRC<70				GROUP 3 REDs			
	mean	SD	median	skew	mean	SD	median	skew	mean	SD	median	skew
SCL-90-R-SOM	62.5	10.35	52	0.340	47.8	9.64	47	0.843	55.6	12.09	54	0.105
SCL-90-R-O-C	59.7	12.05	54	0.126	48.9	9.27	50	0.581	59.4	12.24	60	-0.433
SCL-90-R-I-S	59.4	10.46	55	0.063	48.5	10.44	48	0.612	60.9	11.69	63	-0.484
SCL-90-R-DEP	62.2	9.47	56	-0.042	50.1	9.96	52	0.415	62.6	11.89	66	-0.462
SCL-90-R-ANX	60.7	11.73	55	0.191	48.3	8.66	50	0.751	60.5	12.86	63	-0.437
SCL-90-R-HOS	60.6	12.46	47	0.689	47.4	9.91	45	1.098	52.1	12.16	48	0.632
SCL-90-R-PHOB	59.6	12.44	54	0.511	50.9	9.75	48	0.947	58.6	12.23	57	0.074
SCL-90-R-PAR	57.1	13.53	52	0.180	48.9	9.75	49	0.549	55.0	11.34	54	0.069
SCL-90-R-PSY	59.7	11.17	53	0.046	47.9	8.93	47	0.954	58.9	10.74	58	0.055
SCL-90-R-GSI	62.6	11.59	56	0.092	48.6	9.31	50	0.676	60.9	12.26	62	-0.410

Abbreviations: IBD, Inflammatory bowel disease; EDRC, Eating Disorder Risk Composite; BMI, Body Mass Index; REDs, restrictive eating disorder; SD, standard deviation; skew, skewness; SCL-90-R, Symptom Checklist-90–Revised; SOM, Somatization; O-C, Obsessive–Compulsive; I-S, Interpersonal Sensitivity; DEP, Depression; ANX, Anxiety; HOS, Hostility; PHOB, Phobic anxiety; PAR, Paranoid ideation; PSY, Psychoticism; GSI, Global Severity Index.
